# Systematic and Cell Type-Specific Telomere Length Changes in Subsets of Lymphocytes

**DOI:** 10.1155/2016/5371050

**Published:** 2016-02-10

**Authors:** Jue Lin, Joshua Cheon, Rashida Brown, Michael Coccia, Eli Puterman, Kirstin Aschbacher, Elizabeth Sinclair, Elissa Epel, Elizabeth H. Blackburn

**Affiliations:** ^1^Department of Biochemistry and Biophysics, University of California San Francisco, San Francisco, CA 94143, USA; ^2^Division of Epidemiology, University of California Berkeley, Berkeley, CA 94720, USA; ^3^Department of Psychiatry, University of California San Francisco, San Francisco, CA 94118, USA; ^4^School of Kinesiology, University of British Columbia, Vancouver, BC, Canada V6T 1Z1; ^5^Core Immunology Lab, Division of Experimental Medicine, University of California San Francisco, San Francisco, CA 94110, USA

## Abstract

Telomeres, the protective DNA-protein complexes at the ends of linear chromosomes, are important for genome stability. Leukocyte or peripheral blood mononuclear cell (PBMC) telomere length is a potential biomarker for human aging that integrates genetic, environmental, and lifestyle factors and is associated with mortality and risks for major diseases. However, only a limited number of studies have examined longitudinal changes of telomere length and few have reported data on sorted circulating immune cells. We examined the average telomere length (TL) in CD4+, CD8+CD28+, and CD8+CD28− T cells, B cells, and PBMCs, cross-sectionally and longitudinally, in a cohort of premenopausal women. We report that TL changes over 18 months were correlated among these three T cell types within the same participant. Additionally, PBMC TL change was also correlated with those of all three T cell types, and B cells. The rate of shortening for B cells was significantly greater than for the three T cell types. CD8+CD28− cells, despite having the shortest TL, showed significantly more rapid attrition when compared to CD8+CD28+ T cells. These results suggest systematically coordinated, yet cell type-specific responses to factors and pathways contribute to telomere length regulation.

## 1. Introduction

Telomeres are the DNA-protein complexes at the end of linear chromosomes that are important for genome stability and integrity [1]. The attrition of telomeric DNA can be counteracted by the action of telomerase [2]. Telomere shortening is a manifestation of progression toward cellular damage that can contribute to organismal aging [3]. Human leukocyte telomere length decreases as people age [4] and is determined by multiple inputs including genetic, environmental, and lifestyle factors and disease status [5, 6]. Telomere length is cross-sectionally associated with or predictive of early onset of several chronic diseases [7–11]. The cellular signals and pathways that determine telomere length changes are largely unknown, but several have been proposed. Telomerase, the enzyme that extends telomeric DNA, preferentially acts on short telomeres [12] in cultured cells. In several clinical studies, high telomerase in unstimulated PBMC in combination with short telomere length [13–18] is cross-sectionally associated with worse outcomes, which has led to the yet untested possibility that telomerase may be elevated as a compensatory mechanism in response to short telomeres.

An important question in the field of immune cell telomere research is to understand the extent to which systemic factors may contribute to TL shortening affecting many immune cell subsets, or whether TL shortening rates need to be evaluated separately for each cell subset. If cell-specific TL shortening predominates, this would shift the emphasis away from common upstream factors and toward cell-specific metrics and pathways.

In fetuses and newborn children [20–25], telomere lengths are similar in different tissues, whereas, in adults, different tissues exhibit more marked differences in telomere length [19, 23, 25]. These differences likely result from different replication histories, cell type-specific regulatory pathways, and microenvironments in which the cells reside. Lin et al. [26] recently reported that although telomere lengths were correlated within an individual among PBMCs, T cells and B cells, and monocytes, the rates of change of telomere length for PBMCs, T cells, B cells, and monocytes were not correlated. Telomerase activity, changes of lymphocyte composition, and physiological conditions such as elevated blood glucose and IL-6 levels explained most of the age-dependent telomere length attrition, which is 30% of the total telomere attrition variability.

Comparison of TL in different immune cell types in a cohort of postmenopausal women shows that, relative to other circulating immune cell subtypes, B cells have longer telomeres. TL is especially shortened in CD8+CD28− T cells, compared to other T cell types [19]. These senescent memory cells exert negative effects on immune function [27]. However, whether or not longitudinal telomere length changes in these various immune cell types are correlated has not been studied. Information on the longitudinal TL change in these related but distinct immune cell types will shed light on systemic versus cell type-specific telomere regulation and may help identify the most vulnerable immune cell subsets.

## 2. Materials and Methods

### 2.1. Description of the Cohorts

The entire cohort consists of 183 healthy San Francisco Bay Area premenopausal women who were caregiving for their biological child with an autism spectrum disorder (high stress) and matched control women (low stress), who had children free of any diagnoses. The UCSF Institutional Review Board for Human Research approved the study and all participants provided written consent for this study. All 183 participants had peripheral blood mononuclear cells (PBMCs) sorted into CD4+, CD8+CD28+, and CD8+CD28− T cells and B cells at baseline. In addition, 39 participants (20 high stress and 19 low stress based on being the first participants recruited into the study group) were selected to have PBMC sorted into CD4+, CD8+CD28+, and CD8+CD28− T cells and B cells at 18-month follow-up. Analyses by levels of chronic stress are beyond the scope of this paper and will be reported elsewhere. Caregivers and controls did not differ significantly in age [controls: mean (SD) = 41.5 (4.5), caregivers: mean (SD) = 42.3 (5.6), df = 181, *t* = −0.987, and *p* = 0.325] or BMI [controls: mean (SD) = 25.1 (4.7), caregivers: mean (SD) = 25.9 (5.7), df = 179, *t* = −1.016, and *p* = 0.311]. The sample was examined as a whole, since the questions addressed here were independent of age and stress level. The sample was mostly white, with a mean age of 41.9 (range: 24–51) and mean BMI of 25.5 (range: 17.2–45.1) and had completed college (Table 1).

### 2.2. Telomere Length Assay

Peripheral blood mononuclear cells were purified by Ficoll gradient and sorted into CD4+, CD8+CD28+, and CD8+CD28− T cells and B cells as described earlier [19]. Sorted cells were stored as dry pellets at −80°C and total genomic DNA was purified using the QIAamp® DNA Mini kit (QIAGEN, Hilden, Germany; Cat. number 51104) in batches. DNA was stored at −80°C for batch telomere length measurement. DNA was extracted within 6 months prior to the assay time.

The telomere length assay is adapted from the published original method by Cawthon [19, 28]. The telomere thermal cycling profile consists of cycling for T (telomeric) PCR: 96°C for 1 minute, denaturing at 96°C for 1 second, and annealing/extending at 54°C for 60 seconds, with fluorescence data collection, 30 cycles; cycling for S (single-copy gene) PCR: 96°C for 1 minute, denaturing at 95°C for 15 seconds, annealing at 58°C for 1 second, and extending at 72°C for 20 seconds, 8 cycles; followed by denaturing at 96°C for 1 second, annealing at 58°C for 1 second, extending at 72°C for 20 seconds, and holding at 83°C for 5 seconds with data collection, 35 cycles.

The primers for the telomere PCR are* tel1b* [5′-CGGTTT(GTTTGG)_5_GTT-3′], used at a final concentration of 100 nM, and* tel2b* [5′-GGCTTG(CCTTAC)_5_CCT-3′], used at a final concentration of 900 nM. The primers for the single-copy gene (human beta-globin) PCR are* hbg1* [5′-GCTTCTGACACAACTGTGTTCACTAGC-3′], used at a final concentration of 300 nM, and* hbg2* [5′-CACCAACTTCATCCACGTTCACC-3′], used at a final concentration of 700 nM. The final reaction mix contains 20 mM Tris-HCl, pH 8.4; 50 mM KCl; 200 *μ*M each dNTP; 1% DMSO; 0.4x Syber Green I; 22 ng* E. coli* DNA; 0.4 units of platinum Taq DNA polymerase (Invitrogen Inc.); and 2–10 ng of genomic DNA in a 11 *μ*L reaction. Tubes containing 26, 8.75, 2.9, 0.97, 0.324, and 0.108 ng of a reference DNA (from Hela cancer cells) are included in each PCR run so that the quantity of targeted templates in each research sample can be determined relative to the reference DNA sample by the standard curve method. The same reference DNA was used for all PCR runs.

To control for interassay variability, 8 control DNA samples are included in each run. In each batch, the T/S ratio of each control DNA is divided by the average T/S for the same DNA from 10 runs to get a normalizing factor. This is done for all 8 samples and the average normalizing factor for all 8 samples is used to correct the participant DNA samples to get the final T/S ratio. The T/S ratio for each sample was measured twice. When the duplicate T/S value and the initial value vary by more than 7%, the sample was run the third time and the two closest values were reported. The average coefficient of variation (CV) of this study is 2.3% (±1.8%).

To convert the T/S ratios to base-pairs, the T/S ratios of a set of genomic DNA samples from the human fibroblast primary cell line IMR90 at different population doublings, as well as with the telomerase protein subunit gene (hTERT) transfected into a lentiviral construct, were determined. The mean TRF length from these DNA samples was measured using Southern blot analysis, and the slope of the plot of mean TRF length versus T/S for these samples served as the conversion factor for calculation of telomere length in base-pairs from the T/S ratio. The equation for conversion from T/S ratio to base-pairs is base-pairs = 3274 + 2413 *∗* (T/S).

### 2.3. Telomerase Activity Assay

Gel-TRAP assays were performed by the Telomerase Repeat Amplification Protocol (TRAP) using a commercial kit (TRAPeze Telomerase Detection Kit, Millipore) with modifications [19, 28]. Peripheral blood mononuclear cells were purified by Ficoll gradient and then sorted into CD4+, CD8+CD28+, and CD8+CD28− T cells and B cells as described earlier [19]. 5 × 10^5^–1 × 10^6^ cells per sample were pelleted and stored as dry pellets at −80°C. Cell pellets were lysed with 1XCHAPS buffer as directed by the manual for the TRAPeze kit. An extract corresponding to 5000 cells/*μ*L was made and stored at −80°C and assayed in batches. All the cell subtypes from the same individual were assayed in the same batch. The reaction was carried out according to the TRAPeze kit manual and run on an 8% polyacrylamide-8M urea sequencing gel. The gel was exposed to a phosphorimager plate overnight and scanned on a Typhoon 8600 Imager (GE Healthcare, Piscataway, NJ). The 293T cancer cell line was used as a positive telomerase activity control and standard. Telomerase activity was expressed as equivalent of number of 293T cells. Telomerase activity was quantified using the software ImageQuant 5.2 (GE Healthcare, Piscataway, NJ). Briefly, signals from the product ladders on the gels were added and normalized against the signal from the internal control band for the same lane to get the product/internal control value. For each telomerase activity assay reaction, the product/internal value was divided by the product/internal control value from twenty 293T cells and then multiplied by 20 to obtain the final telomerase activity units, defined as 1 unit = the amount of product from one 293T cell/10,000 immune cells. The average intra-assay variability of PBMC samples (*N* = 6, assayed in triplicate) was 8% and the interassay variability of PBMC samples (*N* = 24, assayed on 2 different days) was 6.7%.

### 2.4. Statistical Analysis

All analyses of TL and TL change were performed on the sample providing both baseline and follow-up data (*N* = 39). Data were complete across all cell types at both baseline and follow-up. Examination of TL distributions within cell types revealed skewness greater than ±1; subsequently, all data were natural log-transformed providing skewness less than 1. No outliers beyond ±4 standard deviations were observed; thus, all values were retained for analysis.

Partial correlations were used to examine cross-sectional relations in TL among CD4+, CD8+CD28+, and CD8+CD28− T cells and B cells, separately, for baseline and follow-up data.

Next, analysis of TL at baseline with TL change (calculated as TL at baseline subtracted from TL at follow-up) revealed significant relations between baseline and shortening. Thus, following Verhulst et al. [29], adjusted TL change values were calculated to correct for apparent regression-to-the-mean (RTM). Finally, the extent of TL change among cell types was compared using a series of paired *t*-tests for each combination of cell types. A Bonferroni correction was used to adjust for these multiple comparisons.

## 3. Results and Discussions

### 3.1. Assay Considerations for Longitudinal Telomere Length Measurement

On average, telomere length changes over time are relatively small [4]. This poses a special challenge for longitudinal measurements, as apparent changes could be attributed to either assay variation or real changes, or a combination of both. We took special precautions to implement the following assay design in order to rule out potential batch differences that might confound longitudinal comparison. The assay was performed as batches of maximal 96 samples on a 384-well assay plate, with triplicate well for each sample. DNA samples from PBMC and sorted cells of the same participant for both the baseline and 18-month follow-up visits were always assayed in the same 96-sample batch. This ensured that comparisons of baseline and 18-month data were always within the same batch, thus eliminating possible systematic biases due to batch difference. In addition, we used one single lot for each reagent for the entire study to eliminate possible batch difference due to different reagent lots.

### 3.2. Telomere Length Change in Sorted Lymphocyte Subtypes within Each Subject Is Correlated

**Figure 1:**
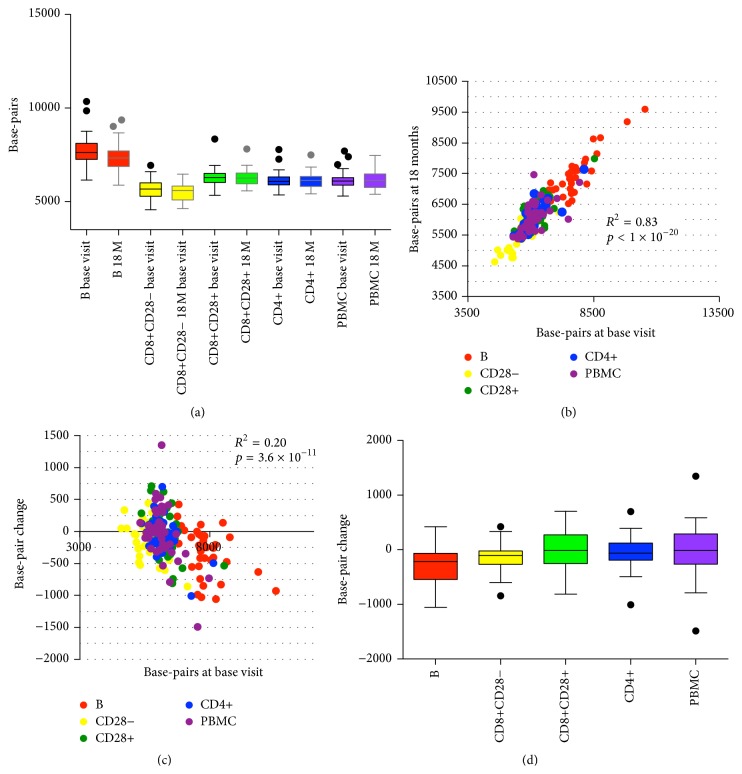
Telomere length in sorted cells and PBMCs. Box and whisker plots display interquartile range, median values, and maximum and minimum values. Telomere length is expressed as basepairs as described earlier [19]. (a) Telomere length in B cells, CD4+, CD8+CD28+, and CD8+CD28− T cells, and PBMCs at base visit and 18 month visit. (b) Telomere length of the base visit and that of 18-month visit from the same cell type of the same individual are highly correlated. The graph includes telomere length from PBMC, CD4+, CD8+CD28+, and CD8+CD28− T cells and B cells from 39 subjects. (c) Telomere length change from base visit to 18 months is negatively correlated with telomere length at base visit. (d) Telomere length change from base visit to 18 months in total PBMC, CD4+, CD8+CD28+, and CD8+CD28− T cells and B cells. *N* = 39 for each cell type.

Earlier work by our group showed that telomere length of total PBMCs and sorted CD4+, CD8+CD28+, and CD8+CD28− T cells and B cells of each subject were correlated. This finding, made in a cohort of postmenopausal women (average age ± SD = 61 ± 8.3) [19], was replicated in the current cohort of premenopausal women. We found that telomere length measurements of CD4+, CD8+CD28+, and CD8+CD28− T cell and B cell populations within each subject were correlated and were also significantly correlated with PBMC telomere length for both baseline and 18-month follow-up visits with the exception of CD8+CD28+ and CD8+CD28− cells at baseline visit (Figure 1(a), Tables 2 and 3); Tables S1 and S2 show column statistics (see Supplementary Material available online at http://dx.doi.org/10.1155/2016/5371050). Also, consistent with earlier results, we found that, of the 5 types examined, B cells exhibited the longest mean telomere length, while CD8+CD28− T cells had the shortest mean telomere length (Figure 1(b) and Table S3).

Multiple previous reports of longitudinal changes in mean telomere length showed a baseline effect, where change in telomere length was negatively correlated with the baseline measurement [30–38]. This phenomenon was also observed for each cell subtype in our cohort, with the correlation in B, CD8+CD28+, and PBMC cells reaching statistical significance (Table S4). One possible explanation of this baseline effect is proposed by Verhulst et al. as regression to the mean due to assay error [29]. To address this, we analyzed the telomere length data after correction for regression to the mean using a regression-to-the-mean (RTM) adjustment approach proposed by Verhulst et al. [29]. The baseline effect disappeared after the adjustment indicating that RTM contributed to the baseline effect observed in this data set (Table S4). We therefore analyzed the telomere length data both as unadjusted and RTM-adjusted.

Cross-sectionally, at both baseline and at 18 M, telomere lengths of all the cell types from the same subject were strongly correlated with the exception of CD8+CD28+ and CD8+CD28− cells at baseline visit (Figure 1(b), Table S5). Interestingly, we found that telomere length changes were also correlated within the same subject. In the RTM-adjusted analysis, within the T cell subtypes, changes of TL in CD4+, CD8+CD28+, and CD8+CD28− were all correlated. In contrast, TL change in B cells was not correlated with the three T cell subtypes. TL change in PBMC was correlated with the three T cell types as well as B cells (Table 4). The unadjusted analysis shows a similar pattern, except that the correlation of PBMC with CD8+CD28+, CD8+CD28−, and B cells does not reach statistical significance (Table S6). This finding is consistent with the lineage relationships among these cell types: the three T cell types are more closely related with each other during cell differentiation than B cells. As PBMCs contain T cells and B cells, the correlation of PBMC TL change with the three T cell types and with B cells as well appears to reflect this composition of PBMCs.

### 3.3. Telomere Length Decreases More in B Cells and CD8+CD28− Cells

Overall, telomere length decreased over the 18-month period, as expected (Figure 1(d), Table 5). However, the rate of change differed for different cell types. B cells decreased most (at an average rate of 196 bp per year) and CD8+CD28− telomere length declined the second fastest (90 bp per year). After RTM adjustment, these differences in the rates of telomere length change persisted and even appeared to be slightly stronger: B cells had more telomere attrition compared to CD8+CD28+, CD4+, and PBMC cells (Table 6). Interestingly, although CD28+CD28− cells have shorter telomeres compared to CD8+CD28+ cells, they have a higher attrition rate compared to CD8+CD28+ cells. CD8+CD28− cells have a higher attrition rate compared to CD4, CD8+28+, and PBMCs but this was marginally significant after adjustments for multiple comparisons. This result suggests that B cells and particularly CD8+CD28− T cells (because of their relatively short baseline telomere length) might be more vulnerable to factors that cause telomere shortening.

Our results are consistent with the recent finding by Lin et al. of the rate of telomere changes being distinct for T cells, B cells, and monocytes in any given subject [26]. Notably, our results differ from a recent report by Daniali et al. that telomeres shorten at the same rate in leukocytes, skin, fat, and muscle tissues in a cohort of 87 subjects [39]. The different results from our longitudinal study and Daniali's study may be because their study was based on a cross-sectional study design and, in addition, the cell types examined in Daniali's and our studies are different.

Cross-sectional studies reported telomere length shortening of 15–19 bps in B cells [40, 41]. To our knowledge, ours is the first study that reported longitudinal changes of TL in B cells in vivo. The 196 bp/year telomere length change in B cells in our study is much larger than the cross-sectional reports. One main difference is that half of the participants in this study are caregivers of autistic children, with high levels of perceived stress. Our previous work and those of others have shown that high psychological stress is associated with shorter telomere length. It is possible that the overall high attrition rate seen in B cells in this study is at least partially due to the presence of these high stress participants.

### 3.4. Telomerase Activity at Baseline Does Not Predict Telomere Length Change at 18 Months

**Figure 2:**
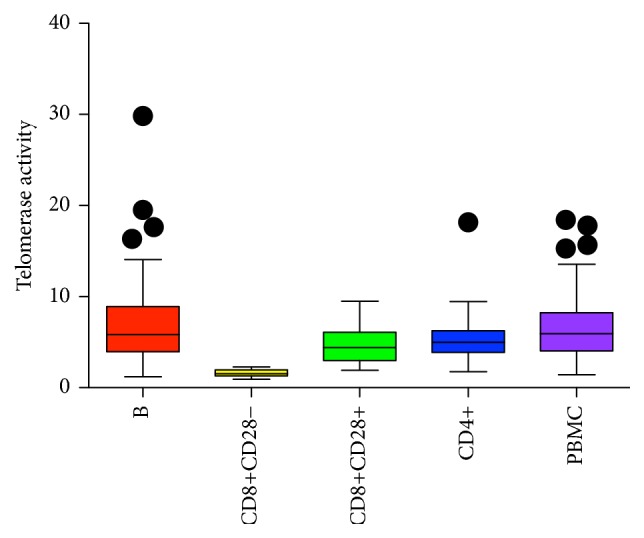
Box and whisker plots display interquartile range, median values, and maximum and minimum values of telomerase activity in B cells, CD4+, CD8+CD28+, and CD8+CD28− T cells, and PBMCs.

Telomere length is under multiple and complex controls. We determined whether baseline telomerase enzymatic activity level, as measured in cell extracts, predicted change in telomere length during the subsequent 18-month period. Such telomerase activity was measured in unstimulated PBMCs, CD4+, CD8+CD28+, and CD8+CD28− T cells and B cells at baseline. Replicating our earlier findings, we found that, in this cohort, within an individual, first, each cell subtype fraction has a characteristic mean telomerase activity level (Figure 2 and Table S1). Comparison of telomerase activity of this cohort to the postmenopausal women cohort [19] shows overall higher telomerase activity levels, consistent with this cohort's younger age. Also, consistent with our earlier results [19], cross-sectionally, there was no correlation between telomere length and telomerase activity within each cell type. Extending our earlier cross-sectional results, the present longitudinal data show that telomerase activity at baseline does not predict telomere length change after 18 months.

Our results are different from those of Lin et al. [26] where telomere attrition in T cells with age was explained by a decline in telomerase activity in a cohort of 216 subjects aged 20–90 years [26]. One possible explanation for the difference between our results and theirs may be due to the narrow age range of our cohort (20 to 50 years old) when compared to theirs. Additionally, our study may be underpowered to detect small effect sizes due to small sample size. Specifically, since CD8+CD28− cells constitute a very small percentage of the total PBMCs, we were only able to obtain enough CD8+CD28− T cells for both telomere length and telomerase activity assays in 11 out of the 39 subjects.

The correlation of TL change in various immune cell types is indicative of underlying common factors, mechanisms, and pathways that contribute to the observed changes. Telomerase, due to its ability to extend telomere length, is proposed to be one such factor. Forced experimental overexpression of telomerase in numerous systems extends telomere length [42]. There are also ample examples during development and disease progression in which telomerase is naturally upregulated, for instance, in B cells in the germinal center, a site where B cells proliferate and mature. This upregulation of telomerase activity is accompanied by telomere length increase [43]. Although logically one would expect telomerase at one point to predict change in telomere length, this had never been tested in normal cells in humans longitudinally. Unlike the results reported by Lin et al. [26], we did not observe predictive relationships between telomerase at baseline and telomere shortening. There could be several reasons why this is the case. Telomerase is under many tight controls, including elaborate cis-regulation of telomerase elongation on telomeric DNA by proteins at the telomeres themselves. Hence, in our cohort and in the cell types examined, many factors other than the measured telomerase activity level may be the predominant forces that determine telomere length change. Secondly, telomerase activity is upregulated in immune cells in response to antigen or mitogen stimulation [44], but we measured the telomerase activity in unstimulated PBMC and sorted cells. It is likely that telomerase activity in stimulated cells is more influential to telomere length change. We note that while we had examined whether telomerase activity at the baseline visit in PBMC, CD4+, CD8+CD28+, and CD8+CD28− T cells and B cells predicted telomere length change in the respective cell types and did not find any association, it is possible that in other cell types telomerase activity is more predictive of telomere length change. For example, as in patients with rare Mendelian telomerase mutations that reduce telomerase activity by half and cause very short telomeres, in the general population, telomerase activity in hematopoietic progenitor cells at the baseline visit would likely be associated with telomere length in the differentiated cells. However, we could not measure the progenitor cells in this study. Finally, it is possible that our study is underpowered to detect associations due to small sample size.

## 4. Conclusions

Circulating immune cells are a mixture of many cell types mainly including T and B cells, within which are various populations of subsets that are developmentally related to one another with shared, yet distinctive, signaling pathways. As these cells are exposed to the same biochemical factors in circulation that impact telomere length change, the rate of telomere length change therefore is the result of cell type-specific intracellular responses to these common external factors.

We show in this study that telomere length changes in CD4+, CD8+CD28+, and CD8+CD28− T cells and B cells from the same participant are correlated, suggesting systematic responses to biochemical factors that impact telomere length. Yet, the rates of telomere length change differ for different immune cell types, indicating cell type-specific responses. Further studies are needed to discover what these factors are, how they exert cell type-specific influences on telomere length change, and how the relationship between these factors and telomere length contributes to disease and risks. These discoveries could have implications for how we understand changes in mixed cell groups. Most studies examine whole blood telomere length and some examine PBMC telomere length. Few studies we are aware of have examined CD8+CD28− change over time, probably because of the expense and effort required. Whole blood consists of a very high percentage of granulocytes, so any greater rate of attrition in presenescent cells (CD8+CD28−) will likely have little influence on the average change in TL. While PBMC telomere length change may be a more sensitive marker of replicative senescence for CD8+ T cells than whole blood, the correlations suggest that CD8+CD28− cells are not showing any greater influence than other cell types on the PBMC change value, which is not surprising given that the CD8+CD28− T cells constitute only a very small percentage of the total PBMCs.

## Supplementary Material

Table S1 provides the means and SD's of telomere length and telomerase activity for the 39 subjects with longitudinal telomere length data (N=39).
Table S2 provides the means and SD's of telomere length and telomerase activity of the entire cohort at base visit.
Table S3 compares telomere length of different cell types from the same individual at baseline visit and 18 month follow-up visit.
Table S4 shows that after regression to the mean adjustment, baseline TL and TL change are not correlated.
Table S5 shows high correlation between baseline TL vs. 18 month TL in all cell types.
Table S6 shows correlation of telomere length change among different cell types without correction for regression to the mean (RTM).

## Figures and Tables

**Table 1:** Participant demographics from the current study^*∗*^.

Characteristic	Mean (SD)/%
Age	41.9 (5.1)
BMI (kg/m^2^)	25.5 (5.2)
Education, years	16.9 (2.1)
Income ($1,000)	142.2 (53.4)
Race/ethnicity (%)	
Caucasian	77.6
Black	3.3
Hispanic or Latina	7.7
Asian, Pacific Islander, or Native American	10.8

^a^The number of participants ranges from 179 to 183.

**Table 2:** Correlation of telomere length among different cell types at the base visit (*N* = 39).

*r* (Pearson)	B	CD8+CD28−	CD8+CD28+	CD4+	PBMC
B	—	0.414^*∗∗*^	0.492^*∗∗*^	0.614^*∗∗∗∗*^	0.544^*∗∗∗*^
CD8+CD28−		—	0.074	0.322^*∗*^	0.336^*∗*^
CD8+CD28+			—	0.796^*∗∗∗∗*^	0.572^*∗∗∗*^
CD4+				—	0.834^*∗∗∗∗*^

^*∗∗∗∗*^ *p* < 0.0001.

^*∗∗∗*^ *p* < 0.001.

^*∗∗*^ *p* < 0.01.

^*∗*^ *p* < 0.05.

T/S ratios were natural log-transformed and adjusted for age.

**Table 3:** Correlation of telomere length among different cell types at the 18-month visit (*N* = 39).

*r* (Pearson)	B	CD8+CD28−	CD8+CD28+	CD4+	PBMC
B	—	0.416^*∗∗*^	0.683^*∗∗∗∗*^	0.723^*∗∗∗∗*^	0.713^*∗∗∗∗*^
CD8+CD28−		—	0.500^*∗∗*^	0.401^*∗∗*^	0.436^*∗∗∗*^
CD8+CD28+			—	0.868^*∗∗∗∗*^	0.753^*∗∗∗∗*^
CD4+				—	0.785^*∗∗∗∗*^

^*∗∗∗∗*^ *p* < 0.0001.

^*∗∗∗*^ *p* < 0.001.

^*∗∗*^ *p* < 0.01.

^*∗*^ *p* < 0.05.

T/S ratios were natural log-transformed and adjusted for age.

**Table 4:** Correlation of telomere length change among different cell types (with correction for regression-to-the-mean (RTM)).

*r* (Pearson)	ΔB	ΔCD8+CD28−	ΔCD8+CD28+	ΔCD4+	ΔPBMC
ΔB	—	0.12	0.30	0.27	0.39^*∗*^
ΔCD8+CD28−		—	0.55^*∗∗∗*^	0.46^*∗∗*^	0.34^*∗*^
ΔCD8+CD28+			—	0.65^*∗∗∗*^	0.43^*∗∗*^
ΔCD4+				—	0.50^*∗∗∗*^

^*∗∗∗*^ *p* < 0.001.

^*∗∗*^ *p* < 0.01.

^*∗*^ *p* < 0.05.

T/S ratios were natural log-transformed and adjusted for age.

**Table 5:** Telomere length change in different cell types.

Cell type	ΔTL (T/S ratio)	StdDev
ΔB	−0.067	0.086
ΔCD8+CD28−	−0.056	0.109
ΔCD8+CD28+	0.000	0.108
ΔCD4+	−0.016	0.086
ΔPBMC	−0.007	0.130

Mean TL change and comparisons are calculated from RTM-adjusted data.

T/S ratios from baseline and 18 months were natural log-transformed.

**Table 6:** Comparison of telomere length change in different cell types (adjusted *p* values).

Comparison	*p* (unadjusted)	*p* (RTM-adjusted)
B versus CD8+CD28−	0.6201	0.5998
**B versus CD8+CD28+**	**0.0034**	**0.0008**
**B versus CD4+**	**0.0085**	**0.0038**
**B versus PBMC**	**0.013**	**0.0042**
**CD8+CD28**−** versus CD8+CD28+**	**0.0068**	**0.0013**
**CD8+CD28**−** versus CD4+**	**0.0251**	**0.0185**
**CD8+CD28**−** versus PBMC**	**0.048**	**0.0299**
CD8+CD28+ versus CD4+	0.2986	0.2264
CD8+CD28+ versus PBMC	0.7834	0.7297
CD4+ versus PBMC	0.6568	0.6133

T/S ratio changes from baseline to 18 months were natural log-transformed and paired samples tests were performed. False discovery rate adjusted *p* value of <0.005 is considered significant.
