# Supplementary material for: Systematic and Cell Type-Specific Telomere Length Changes in Subsets of Lymphocytes
Source: J Immunol Res. 2016 Feb 10;2016:5371050. doi: 10.1155/2016/5371050 (PMC4764743; doi:10.1155/2016/5371050)
Supplement: Supplementary file 1 — Table S1 provides the means and SD's of telomere length and telomerase activity for the 39 subjects with longitudinal telomere length data (N=39). Table S2 provides the means and SD's of telomere length and telomerase activity of the entire cohort at base visit. Table S3 compares telomere length of different cell types from the same individual at baseline visit and 18 month follow-up visit. Table S4 shows that after regression to the mean adjustment, baseline TL and TL change are not correlated. Table S5 shows high correlation between baseline TL vs. 18 month TL in all cell types. Table S6 shows correlation of telomere length change among different cell types without correction for regression to the mean (RTM). [file 5371050.f1.docx]

**Supplementary Materials**

**Table S1:** Column statistics of telomere length and telomerase activity (N=39)

|  | Base visit T/S | | 18 month visit T/S | | Base visit TA | |
| --- | --- | --- | --- | --- | --- | --- |
|  | Mean | SD | Mean | SD | Mean | SD |
| B | 1.82 | 0.36 | 1.70 | 0.31 | 7.56 | 5.82 |
| CD8+CD28- | 0.99 | 0.22 | 0.94 | 0.19 | 1.57* | 0.43 |
| CD8+CD28+ | 1.25 | 0.21 | 1.25 | 0.19 | 4.65 | 1.91 |
| CD4+ | 1.20 | 0.20 | 1.18 | 0.18 | 5.41 | 2.78 |
| PBMC | 1.19 | 0.21 | 1.18 | 0.20 | 6.97 | 4.31 |

*N=11. Due to low percentage of CD8+CD28- in PBMC population, not enough cells were obtained from 28 subjects for telomerase activity assay)

**Table S2:** Means and SD’s of telomere length and telomerase activity of the entire cohort at base visit

| Cell type | T/S | | | TA activity | | |
| --- | --- | --- | --- | --- | --- | --- |
|  | N | Mean | SD | N | Mean | SD |
| B | 178 | 1.71 | 0.30 | 149 | 6.44 | 5.81 |
| CD8+CD28- | 176 | 1.02 | 0.25 | 47 | 2.27 | 1.87 |
| CD8+CD28+ | 176 | 1.24 | 0.24 | 163 | 4.66 | 4.94 |
| CD4+ | 178 | 1.21 | 0.20 | 167 | 4.66 | 2.71 |
| PBMC | 179 | 1.17 | 0.20 | 169 | 5.43 | 3.44 |

**Table S3:** Telomere length values from different cell types from the same individual are different at baseline and 18 month followup

| comparison | p | |
| --- | --- | --- |
|  | baseline | 18 month |
| B vs. CD8+CD28- | <0.0001 | <0.0001 |
| B vs. CD8+CD28+ | <0.0001 | <0.0001 |
| B vs. CD4+ | <0.0001 | <0.0001 |
| B vs. PBMC | <0.0001 | <0.0001 |
| CD8+CD28- vs. CD8+CD28+ | <0.0001 | <0.0001 |
| CD8+CD28- vs. CD4+ | <0.0001 | <0.0001 |
| CD8+CD28- vs. PBMC | <0.0001 | <0.0001 |
| CD8+CD28+ vs. CD4+ | 0.045 | <0.0001 |
| CD8+CD28+ vs. PBMC | <0.0001 | 0.003 |
| CD4+ vs. PBMC | <0.0001 | 0.93 |

T/S ratios are natural log transformed and paired T tests are performed. False discovery rate adjusted p value of <0.005 is considered significant.

## Table S4: Regression between Baseline TL vs. TL change after 18 months

|  | Unadjusted | | RTM adjusted | |
| --- | --- | --- | --- | --- |
| Cell type | Standardized beta | p | Standardized beta | p |
| B | -0.35 | 0.035 | 0.1 | 0.57 |
| CD28+CD28- | -0.27 | 0.112 | 0.01 | 0.95 |
| CD28+CD28+ | -0.37 | 0.0295 | -0.08 | 0.66 |
| CD4+ | -0.25 | 0.141 | -0.06 | 0.71 |
| PBMC | -0.38 | 0.020 | -0.07 | 0.70 |

*T/S were natural log transformed and age-adjusted.

## Table S5: Correlation between Baseline TL vs. 18 month TL

| Cell type | r |
| --- | --- |
| B | 0.88 |
| CD28+CD28- | 0.87 |
| CD28+CD28+ | 0.64 |
| CD4+ | 0.81 |
| PBMC | 0.61 |

*T/S were natural log transformed and age-adjusted.

**Table S6:** Correlation of telomere length change among different cell types (without correction for regression to the mean (RTM)

| r (Pearson) | ∆ B | ∆ CD8+CD28- | ∆ CD8+CD28+ | ∆ CD4+ | ∆ PBMC |
| --- | --- | --- | --- | --- | --- |
| ∆ B | - | 0.100 | 0.21 | 0.18 | 0.29 |
| ∆ CD8+CD28- |  | - | 0.42** | 0.44** | 0.29 |
| ∆ CD8+CD28+ |  |  | - | 0.59*** | 0.21 |
| ∆ CD4+ |  |  |  | - | 0.44** |

***p<0.001

**p<0.01

*p<0.05

T/S ratios were natural log transformed and adjusted for age
